# Can Transcranial Electrical Stimulation Facilitate Post-stroke Cognitive Rehabilitation? A Systematic Review and Meta-Analysis

**DOI:** 10.3389/fresc.2022.795737

**Published:** 2022-02-10

**Authors:** Ahsan Khan, Kai Yuan, Shi-Chun Bao, Chun Hang Eden Ti, Abdullah Tariq, Nimra Anjum, Raymond Kai-Yu Tong

**Affiliations:** ^1^Biomedical Engineering Department, The Chinese University of Hong Kong, Hong Kong, China; ^2^National Innovation Center for Advanced Medical Devices, Shenzhen, China; ^3^Department of Electrical Engineering, Pakistan Institute of Engineering and Applied Sciences, Islamabad, Pakistan; ^4^Hong Kong Brain and Mind Institute, The Chinese University of Hong Kong, Hong Kong, China

**Keywords:** cognitive rehabilitation, stroke, transcranial electric stimulation, transcranial direct current simulation, cognitive defcits

## Abstract

**Background:**

Non-invasive brain stimulation methods have been widely utilized in research settings to manipulate and understand the functioning of the human brain. In the last two decades, transcranial electrical stimulation (tES) has opened new doors for treating impairments caused by various neurological disorders. However, tES studies have shown inconsistent results in post-stroke cognitive rehabilitation, and there is no consensus on the effectiveness of tES devices in improving cognitive skills after the onset of stroke.

**Objectives:**

We aim to systematically investigate the efficacy of tES in improving post-stroke global cognition, attention, working memory, executive functions, visual neglect, and verbal fluency. Furthermore, we aim to provide a pathway to an effective use of stimulation paradigms in future studies.

**Methods:**

Preferred reporting items for systematic reviews and meta-analysis (PRISMA) guidelines were followed. Randomized controlled trials (RCTs) were systematically searched in four different databases, including Medline, Embase, Pubmed, and PsychInfo. Studies utilizing any tES methods published in English were considered for inclusion. Standardized mean difference (SMD) for each cognitive domain was used as the primary outcome measure.

**Results:**

The meta-analysis includes 19 studies assessing at least one of the six cognitive domains. Five RCTs studying global cognition, three assessing visual neglect, five evaluating working memory, three assessing attention, and nine studies focusing on aphasia were included for meta-analysis. As informed by the quantitative analysis of the included studies, the results favor the efficacy of tES in acute improvement in aphasic deficits (SMD = 0.34, CI = 0.02–0.67, *p* = 0.04) and attention deficits (SMD = 0.59, CI = −0.05–1.22, *p* = 0.07), however, no improvement was observed in any other cognitive domains.

**Conclusion:**

The results favor the efficacy of tES in an improvement in aphasia and attentive deficits in stroke patients in acute, subacute, and chronic stages. However, the outcome of tES cannot be generalized across cognitive domains. The difference in the stimulation montages and parameters, diverse cognitive batteries, and variable number of training sessions may have contributed to the inconsistency in the outcome. We suggest that in future studies, experimental designs should be further refined, and standardized stimulation protocols should be utilized to better understand the therapeutic effect of stimulation.

## Introduction

Two-thirds of stroke survivors experience a cognitive deficit or decline after the onset of stroke, which hampers functional performance in activities of daily living and results in poor quality of life (1, 2). With an increasing prevalence of stroke in the last two decades, rehabilitation of stroke survivors has been a prime research topic for researchers and healthcare authorities.

Neuroanatomical lesions caused by the stroke on strategically important cognitive areas (such as the hippocampus, medial prefrontal cortex, and cingulate cortex), lesions in the white matter, and cerebral microbleeds mainly contribute to the pathogenesis of post-stroke cognitive impairment (3). Cognition is not a unitary concept; different domains cooperate as a system (4), including attention, executive function, visuospatial ability, memory, and language. Likewise, post-stroke cognitive impairments occur in multiple cognitive domains (5, 6). Different techniques including, cognitive training, brain stimulation, and pharmacological agents, have been applied to promote neural plasticity and improve the cognitive function of stroke survivors (4, 7).

Non-invasive brain stimulation (NIBS) has emerged as a potential rehabilitation tool for brain disorders (8–13) with transcranial magnetic stimulation (TMS) and transcranial electrical stimulation (tES) being the most widely utilized non-invasive methods. The efficacy of TMS in treating depressive disorders paved its approval from the US Food and Drug Administration (FDA) for the treatment of depression (14) and it works on the principle of electromagnetic induction in which magnetic fields are passed into the brain, and they induce electric fields at the targeted regions (15). On the other hand, tES devices have gained particular interest in the last couple of decades due to their non-invasiveness, affordability, safety, and easier accessibility (16, 17), which is the main focus of the current study. The working principle of tES is rather straightforward in which electrical currents are passed in the brain to influence the activity of underlying neurons. TES has been classified into three different types of stimulation methods based on the type of electrical current, i.e., transcranial direct current stimulation (tDCS), transcranial alternating current stimulation (tACS), and transcranial random noise stimulation (tRNS). In tDCS, direct electrical currents are passed in the brain, which either excites or inhibit the neuronal excitability depending on the polarity of the stimulation (18). tACS influences cortical excitability in a frequency-dependent manner (19), while tRNS is a special case of tACS which delivers weak alternating currents at random frequencies (20).

The literature on tES studies is filled with inconsistencies, and there is no definite conclusion on the efficacy of tES interventions in improving cognition (21). From the physiological point of view, the application of tDCS to the brain shifts the resting membrane potential of superficial horizontal intracortical interneurons, which induce changes to spontaneous neuronal excitability (22). The initial membrane potential shifts result in long-term modification of synaptic plasticity by processes similar to long-term potentiation (LTP) and long-term depression (LTD) through the modulation of NMDA receptors (23). There is a general agreement that anodal stimulation increases excitation in the brain while cathodal results in inhibition (18). However, even this generalization could not be replicated in various studies (24–26). The working principle of tACS is fundamentally different from tDCS in a way that it does not influence average membrane potential. In one cycle, one electrode acts as an anode and the other one as a cathode, while in the other cycle, the pattern is reversed, and the average membrane potential is unchanged. The online effects of tACS are induced as a result of entrainment of applied frequencies to the underlying endogenous brain activity (19). Thus, non-invasive electrical brain stimulation tools such as tDCS and tACS can induce alterations in cortical excitability, oscillatory, and non-oscillatory activities, which are the physiological derivates of cognitive processes, such as perception working memory, learning, and long-term memory formation (27–29).

There are several other factors that determine the impact of stimulation on the brain. For example, tES devices have been further classified into conventional and high-definition tES (30). Conventional tES (C-tES) uses a pair of large electrode patches, while High-definition tES (HD-tES) uses a series of ring electrodes. HD-tES has been introduced as an advanced version of C-tES, targeting specific brain regions with higher focality (31). In addition, in the case of tDCS, the return electrode is ideally placed at a location where it does not influence the cortical excitability (32). However, for tACS, there is no distinction between anode and cathode, as electrodes switch their roles in every cycle. Furthermore, the stimulation montage, intensity, and duration play a critical role in determining its impact on the brain (33).

Studies on healthy human participants have repeatedly demonstrated that the stimulation of cognitive brain regions with tDCS or tACS results in the modulation of the brain activity and it often results in modulation of behavior (34–38). However, the effect of these stimulation methods on stroke with cognitive deficits has not been thoroughly investigated. Most of the previous meta-analyses on post-stroke cognitive rehabilitation utilized TMS, and they often focused on aphasic patients only (13, 39–41). In this study, we aim to investigate the efficacy of tES on post-stroke cognitive rehabilitation. We systematically evaluated six cognitive domains, including attention, working memory, executive control, aphasia, and visual neglect following Preferred reporting items for systematic reviews and meta-analysis (PRISMA) guidelines. We further highlight ways to standardize and refine stimulation protocols to improve the effectiveness of tES devices in rehabilitation.

## Materials and Methods

### Protocol Registration

Following PRISMA guidelines, the protocol was registered at Prospective Register of Systematic Review (PROSPERO), under the identification number CRD42021237806.

### Literature Search

Four different databases, including PubMed (Medline), EMBASE, Web of Science, and PsycINFO were searched independently for the studies meeting inclusion criteria. The detailed search criteria, which was a modified version of search criteria for another meta-analysis on cognitive impairment after stroke (42), are provided in Supplementary Materials.

### Inclusion Criteria

Studies with the following criteria were included in the analysis: (1) Randomized controlled trials applying tES for a single session or multiple sessions; (2) Longitudinal or a crossover study design; (3) Ischemic or hemorrhagic stroke; (4) Single/double/triple blinded studies comparing the tES stimulation to a control group receiving sham stimulation or no stimulation. If the intervention was performed with another component, for example cognitive training then intervention plus cognitive training was compared with sham plus cognitive training; (5) Studies reporting sufficient information to compute effect size statistics [i.e., mean and standard deviations or standard errors, exact *F, p, t*, or *z*-values]; (6) Published in an international peer-reviewed journal until 31st December 2020; (7) Published in English language.

### Main Outcomes

The systematic review focussed on six cognitive domains including, global cognition, visual neglect, attention, working memory, executive functions, and verbal fluency. If multiple cognitive tests were used for each cognitive domain, then the test listed as the primary measure was included. If there was no explicit distinction mentioned between the primary and secondary measures, then the test most relevant to the cognitive domain was included. If follow-up measurements are performed after training, then scores immediately after the training were considered for evaluation of the outcome.

### Measurement of Effect Size

The mean difference (MD) or standardized mean difference (SMD) with a confidence interval (CI) of 95% was used to measure the effect size depending on the cognitive batteries used in different studies. If in case standard deviation between pre- and post-training cannot be estimated using the available data then sensitivity analysis was performed on studies by testing the effect of each study at different correlation values. If any of the studies potentially influence the results then it was removed. Two-sided *p* < 0.05 was considered statistically significant.

### Data Extraction

After the identification of studies through the search strategy, duplicate studies were removed using EndNote 20. Following the removal of duplicate studies, titles of the remaining studies were evaluated by Reviewer 1 and Reviewer 2, and studies not meeting the inclusion criteria were eliminated. The second round of evaluation was performed on the abstracts by the same two reviewers. After screening of the titles and abstracts, full articles were assessed by the Reviewer 1 and Reviewer 2. Some articles which did not provide enough information to estimate the effect size or used same population in multiple studies were excluded after qualitative analysis. After the selection of studies, the data, including study design, sample size, participants characteristics, type of intervention, duration, and intensity of tES, and outcomes was extracted by Reviewer 5 and Reviewer 6. Following data extraction, Reviewer 1 performed statistical analysis on the data and was cross-checked by Reviewer 2. Reviewers 3 and 4 assessed methodological quality and the risk of bias in each of the included studies. Reviewer 7 supervised the whole study and was consulted in case of a disagreement about any study.

### Risk of Bias Assessment

To assess the methodological quality and risk of bias, the modified Jadad scale with eight items was utilized (43). Generation of random sequence, description of blinding procedures, description of withdrawals and dropouts, description of inclusion/exclusion criteria, description of adverse effects, and statistical analysis were evaluated against a scale of 8 (range:−2–8), higher scores indicate better research quality.

### Data Analysis

Review Manager 5.4.1 was used for performing a meta-analysis. Hedge's g was utilized to quantify the effect size with a *g* < 0.2 reflecting a small effect size, 0.5 reflecting a medium effect size, and >0.8 reflecting a large effect size. Higgins I^2^ statistic was employed to measure the degree of heterogeneity between the studies. *I*^2^-values of 25, 50, and 75% were considered as low, moderate, and high heterogeneity, respectively. Sensitivity analysis was performed to identify the source/sources of heterogeneity (44).

## Results

### Search Results

**Figure 1:**
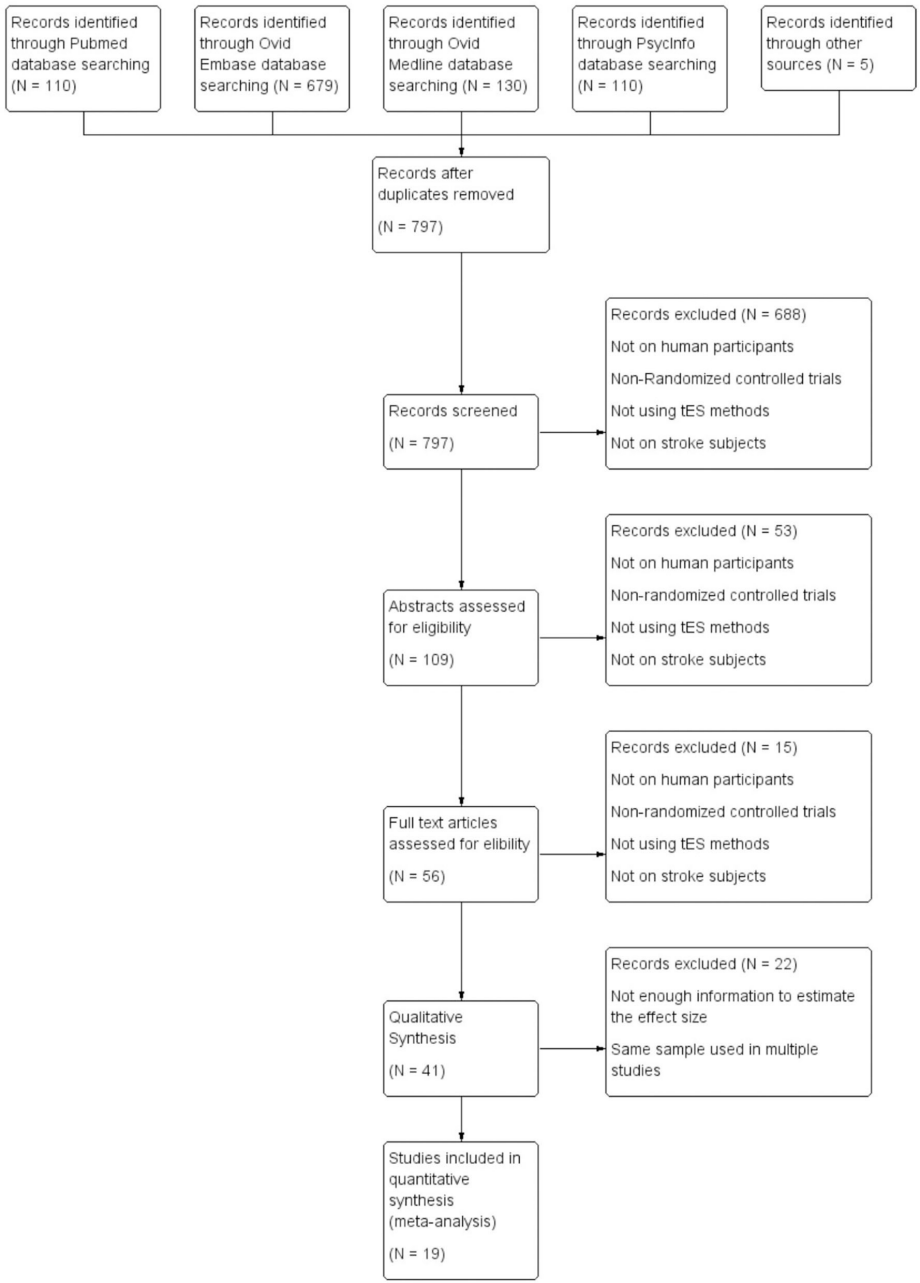
Flow chart of selection of studies.

After the removal of duplicates, titles of the remaining 797 studies were screened and 109 studies were identified with 20 systematic reviews. Systematic reviews were separately assessed for studies meeting inclusion criteria in case they were not identified in the initial search. Abstracts were further screened for inclusion and 56 studies were identified. These studies were evaluated and 19 studies were included for quantitative analysis. Out of these 19 studies, 5 studies evaluated global cognition, 3 studied attention, 5 assesed working memory, 9 studies focused on aphasia, and 3 studies evaluated visual neglect. It should be noted that a few studies evaluated multiple cognitive domains and they have been analyzed separately in each of the domains. Among the 19 studies included in the quantitative analysis, 13 studies provided information regarding the type of stroke (ischemic or hemorrhagic), and 75.7% of the subjects suffered from ischemic stroke; 12 studies reported the lesion location (subcortical, cortical or mixed) and 35.0% of the subjects had pure subcortical lesions; 18 studies reported the gender information and 62.3% of the subjects were male; 17 studies offered information regarding the time after stroke onset, with 9 studies on chronic stroke subjects, 3 studies recruited subacute subjects, 4 studies recruited both subacute and chronic subjects, and 1 study recruited acute and subacute subjects; 16 out of 19 studies were conducted in Asia (*n* = 8) and Europe (*n* = 8), 2 studies were conducted in North America and 1 study was conducted in Africa. Flow chart of the selection process is shown in Figure 1. All the included studies are listed in Table 1.

**Table 1:** Characteristics of the included studies.

**References**	**Design**	**Participants**	**Stroke type**	**Stimulation parameters**	**Stimulation type**	**Target electrode location**	**Reference electrode location**	**Cognitive domains**
Kang et al. (45)	Cross-Over	*N_*exp*_* = 10 *N_*sham*_* = 10 Age_exp_ = 69.9 ± 3 Age_sham_ = 69.9 ± 3	Subacute and chronic	Intensity: 2 mA Duration: 20 min	Conventional tDCS	Left DLPFC	Contralateral supraorbital region	Attention
Jo et al. (46)	Cross-Over	*N_*exp*_* = 10 *N_*sham*_* = 10 Age_exp_ = 47.9 ± 8.9 Age_sham_ = 47.9 ± 8.9	Subacute and chronic	Intensity: 2 mA Duration: 30 min	Conventional tDCS	Left DLPFC	Right supraorbital area	Working memory
Fiori et al. (47)	Cross-Over	*N_*exp*_* = 3 *N_*sham*_ =* 3 Age_exp_ = 65, 74, 45 Age_sham_ = 65, 74, 45	Chronic	Intensity: 1 mA Duration: 20 min	Conventional tDCS	Left Wernicke's area	Contralateral fronto-polar cortex	Aphasia
Kang et al. (48)	Cross-Over	*N_*exp*_* = 10 *N_*sham*_* = 10 Age_exp_ = 61.9 ± 2.7 Age_sham_ = 61.9 ± 2.7	Chronic	Intensity: 2 mA Duration: 20 min	Conventional tDCS	Right Broca's homolog area	Left supraorbital area	Aphasia
Sunwoo et al. (49)	Cross-Over	*N_*exp*_* = 10 *N_*sham*_* = 10 Age_exp_ = 62.6 ± 13.3 Age_sham_ = 62.6 ± 13.3	Subacte and chronic	Intensity_dual_: 2 mA Intensity_single_: 1 mA Duration: 20 min	Conventional tDCS	Right PPC, right supraorbital area	Left supraorbital area, Left PPC	Visual neglect
Marangolo et al. (50)	Cross-Over	*N_*exp*_* = 7 *N_*sham*_* = 7 Age_exp_ = 46–77 Age_sham_ = 46–77	Chronic	Intensity: 1 mA Duration: 20 min	Conventional tDCS	Wernicke's area, Broca's area	Contralateral fronto-polar cortex	Aphasia
Volpato et al. (39)	Cross-Over	*N_*exp*_* = 8 *N_*sham*_* = 8 Age_exp_ = 42–70 Age_sham_ = 42-70	Chronic	Intensity: 2 mA Duration: 20 min	Conventional tDCS	Broca's area	Contralateral supraorbital area	Aphasia
Park et al. (51)	Parallel	*N_*exp*_* = 6 *N_*sham*_* = 5 Age_exp_ = 65.3 ± 14.3 Age_sham_ = 66.0 ± 10.8	Subacute	Intensity: 2 mA Duration: 30 min	Conventional tDCS	Bilateral prefrontal cortex	Non-dominant arm	Global cognition
Cotelli et al. (52)	Parallel	N_exp_ = 8 N_sham_ = 8 Age_exp_ = 63.4 ± 6.8 Age_sham_ = 70.4 ± 6.8	NS	Intensity:2 mA Duration: 25 min	Conventional tDCS	left DLPFC	Right arm	Aphasia
Yun et al. (40)	Parallel	*N_*LFTAS*_* = 15 *N_*RFTAS*_* = 15 *N_*sham*_* = 15 Age_LFTAS_ = 60.9 ± 12.9 Age_RFTAS_ = 58.9 ± 15 Age_sham_ = 68.5 ± 14.6	Acute and subacute	Intensity: 2 mA Duration: 30 min	Conventional tDCS	Left FTAS, Left anterior temporal lobe; Right FTAS, right anterior temporal lobe	Not mentioned	Global cognition
Ladavas et al. (53)	Parallel	*N_*anodal*_* = 11 *N_*cathodal*_* =8 *N_*sham*_* = 11 Age_anodal_ = 46-78 Age_cathodal_ = 59–78 Age_sham_ = 49–78	Subacute and Chronic	Intensity: 2 mA Duration: 20 min	Conventional tDCS	PPC	Anodel: left supraorbital region Cathodal: right supraorbital region	Visual neglect
Bang and Bong (54)	Parallel	*N_*exp*_* = 6 *N_*sham*_* = 6 Age_exp_ = 66.0 ± 4.2 Age_sham_ = 65.6 ± 4.7	Subacute	Intensity: 1 mA Duration: 20 min	Conventional tDCS	Right PPC	Left supraorbital area	Visual neglect
Andre et al. (55)	Parallel	*N_*exp*_* = 13 *N_*sham*_* = 8 Age_exp_ = 63–94 Age_sham_ = 63–94	NS	Intensity: 2 mA Duration: 20 min	Conventional tDCS	Left DLPFC	contralateral supraorbital area	Working memory, global cognition, attention,
Meinzer et al. (56)	Parallel	*N_*exp*_* = 13 *N_*sham*_* = 13 Age_exp_ = 38-77 Age_sham_ = 41-78	Chronic	Intensity: 1 mA Duration: 20 min	Conventional tDCS	Left M1	Right supraorbital region	Aphasia
Darkow et al. (57)	Cross-over	*N_*exp*_* = 16 *N_*sham*_* = 16 Age_exp_ = 56.7 ± 10.1 Age_sham_ = 56.7 ± 10.1	Chronic	Intensity: 1 mA Duration: 20 min	Conventional tDCS	Left M1	Right supraorbital area	Aphasia
Kazuta et al. (58)	Cross-over	*N_*exp*_* = 12 *N_*sham*_* = 12 Age_exp_ = 71.5 ± 7.4 Age_sham_ = 71.5 ± 7.4	Chronic	Intensity: 2 mA Duration: 9.5 min	Conventional tDCS	Left temporoparietal area	Right supraorbital region	Working memory
Norise et al. (59)	Partial Crossover	*N_*exp*_* = 9 *N_*sham*_* = 9 Age_exp_ = 57.3 ± 4.6 Age_sham_ = 57.3 ± 4.6	Chronic	Intensity: 2 mA Duration: 20 min	Conventional tDCS	Left frontal lobe, right frontal lobe	Contralateral mastoid	Aphasia
Shaker et al. (60)	Parallel	*N_*exp*_* = 20 *N_*sham*_* = 20 Age_exp_ = 54.45 ± 4.68 Age_sham_ = 53.05 ± 6.32	Chronic	Intensity: 2 mA Duration: 30 min	Conventional tDCS	Right and Left DLPFC	Contralateral supraorbital area	Global cognition, attention, working memory
Zumbansen et al. (41)	Parallel	*N_*exp*_* = 24 *N_*sham*_* = 19 Age_exp_ = 65.3 ± 13.2 Age_sham_ = 67.4 ± 11.7	Subacute	Intensity: 2 mA Duration: 45 min	Conventional tDCS	Right pars triangularis	Forehead over the right eye	Aphasia

*NS, not specified; DLPFC, dorsolateral prefrontal cortex; PPC, posterior parietal cortex; M1, primary motor cortex; FTAS, fronto-temporal anodal stimulation*.

### Global Cognitive Functions

**Figure 2:**
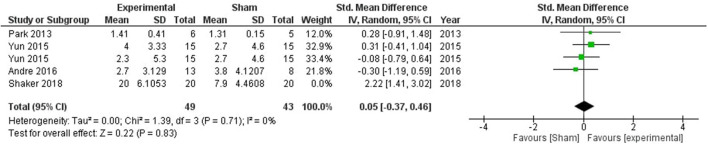
Comparison of the effects of active transcranial stimulation and sham stimulation interventions on global cognition. The results did not provide evidence for tDCS efficacy in promoting global cognition (SMD = 0.05; 95% CI = −0.37–0.46; *p* = 0.83).

Global cognitive deficits are common after stroke which significantly affect the activities of daily living. One of the pioneering studies by Park et al. (51) combined computer-assisted cognitive rehabilitation (CACR) and tDCS five times a week for 30 min a session in a bid to improve global cognitive functions. Patients were evaluated using Korean Mini-Mental State Examination (K-MMSE) and Seoul Computerized Neuropsychological Test (SCNT). However, no significant difference was observed between the experimental and sham groups in K-MMSE scores. The SCNT item on the auditory continuous performance test (CPT) was significantly higher in the tDCS group as compared with the control group. Another noteworthy study by Yun et al. (40) explored the impact of anodal tDCS on left and right fronto-temporal brain regions. Participants were divided into 3 groups with 15 patients in each group i.e., left fronto-temporal anodal stimulation (FTAS), right FTAS, and sham. Fifteen sessions of 2 mA tDCS was delivered for 30 min, However, no significant difference was observed in any of the treatment groups. Andre et al. (55) explored the effect of stimulation of the left dorsolateral prefrontal cortex (DLPFC) by applying four 20 min sessions of 2 mA anodal or sham at-home tDCS, however, no stimulation-induced beneficial effect was observed. Another study conducted by Shaker et al. (60) provided RehaCom training to stroke subjects along with anodal or sham stimulation targeted at left DLPFC. The treatment lasted for a month with three sessions on alternating days in each week. Participants received 30 min of stimulation followed by 30 min of RehaCom training in the stimulation group, while the sham group only received 5 s of stimulation. A significant improvement in global cognitive functions was observed in the experimental group. Meta-analysis of all the mentioned studies did not show any beneficial effect of stimulation on global cognitive functioning (SMD = 0.31, CI = −0.27–0.89, *p* = 0.30) with high heterogeneity (*I*^2^ = 61%; Supplementary Figure 1). After sensitivity analysis, one study by Shaker et al. (60) was removed from the quantitative analysis which reduced the heterogeneity to 0, however, the overall effect of stimulation was non-significant (SMD = 0.02, CI = −0.39–0.43, *p* = 0.93; Figure 2).

### Attention

Attention decline after stroke is one of the most common cognitive deficits which hampers the rehabilitation process, reduces physical function recovery, and increases dependence on others during the activities of daily life. A study by Kang et al. (45) administered single session anodal stimulations on the left DLPFC for 20 min with a current intensity of 2 mA. tDCS led to a significant improvement in a computerized Go/No-Go response accuracy at 1 h post-stimulation relative to baseline, and this improvement was maintained until 3 h post-stimulation. Andre et al. (55) studied multiple session anodal stimulation effect on left DLPFC using a Go/No-Go task. After four 20 min sessions of 2 mA current intensity, an improvement in the reaction time in the active tDCS group was observed. Shaker et al. (60) in their study involving Rehacom training reported a significant improvement in attentive abilities for the experimental group.

**Figure 3:**
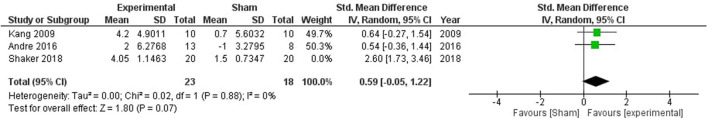
Comparison of the effects of active transcranial stimulation and sham stimulation interventions on attention. One study was excluded after sensitivity analysis. The results favored the efficacy of stimulation in facilitating attentive abilities (SMD = 0.59, CI = −0.05–1.22, *p* = 0.07).

Meta-analysis of the included studies showed a significant impact of stimulation on attentive abilities (SMD = 1.26, CI = −0.07–2.60, *p* = 0.06) with high heterogeneity (*I*^2^ = 78%) (Supplementary Figure 2). One study by Shaker et al. (60) was removed which reduced the heterogeneity to 0%, and a significant overall effect (SMD = 0.59, CI = −0.05–1.22, *p* = 0.07; Figure 3).

### Working Memory

Working memory impairments are very common after stroke. Jo et al. (46) examined whether one session of anodal tDCS on left DLPFC can influence the working memory of stroke patients. A current of 2 mA was administered for 30 min on 10 stroke subjects in a crossover study design. Results suggested that anodal tDCS can enhance working Memory performance by improving accuracy, however, no change in reaction time was observed. Leśniak et al. (61) also explored the impact of anodal tDCS on verbal learning and memory performance by utilizing Rey Auditory Verbal Learning Test (RAVLT), however, no significant improvement was observed after 15 sessions of training. Andre et al. (55) investigated the effects of anodal tDCS on the visual short-term memory of patients using the picture-naming task (PNT) and a slight improvement was observed after 4 sessions tDCS. In 2017 Kazuta et al. (58) studied the effect of anodal-tDCS on the audio verbal memory performance of 12 stroke patients using the RAVLT test. Shaker et al. (60) in their study involving RehaCom training reported a significant improvement in the figural working memory performance.

**Figure 4:**
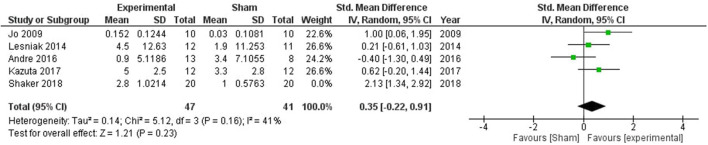
Comparison of the effects of active transcranial stimulation and sham stimulation interventions on working memory. One study was excluded after sensitivity analysis. No stimulation related improvement was observed in the working memory (SMD = 0.35; 95% CI = −0.22–0.91; *p* = 0.23).

Meta-analysis showed a significant stimulation impact on attentive abilities (SMD = 0.72, CI = −0.14–1.57, *p* = 0.10*)* with high heterogeneity (*I*^2^ = 80%) (Supplementary Figure 3). One study by Shaker et al. (60) was removed after sensitivity analysis which reduced the heterogeneity to 41%, and the overall effect (SMD = 0.35, CI = −0.22–0.91, *p* = 0.23; Figure 4).

### Executive Functions

Executive functions are defined as a set of cognitive skills that work altogether to exhibit a goal directed behavior. The literature on direct evidence of stimulation's impact on executive functions is very limited. We could not identify any study specifically focusing on executive functions in stroke patients.

### Aphasia

Aphasia is a language disorder that affects the production or comprehension of speech that often occurs after a stroke. Fiori et al. (62) studied the impact of anodal stimulation in aphasic subjects by targeting left Wernicke's Area which is involved in language production and comprehension. In each stimulation condition, the treatment was carried out for five consecutive days in 1 week and a significant improvement was observed in the stimulation group. Kang et.al (48) reported a significant improvement in a picture naming task followed by stimulation of Broca's homolog area. In another study (39), chronic aphasics underwent 2 weeks of offline anodal tDCS on Broca's area and 2 weeks of sham stimulation as a control condition. However, no significant interaction was observed. A 2013 study by Marangolo et al. (50) studied the impact of anodal stimulation over Broca's area and Wernicke's area in aphasic patients. Results indicated a significant improvement in the stimulation group as compared to the sham group. Broca's area results have been included in the meta-analysis as its more relevant to language disorders. In another study (52), DLPFC was stimulated in 16 aphasics during speech therapy with either anodal or sham stimulation. Aachener Aphasic Test accuracy showed a significant improvement in the anodal group as compared to the sham group. Marcus Meinzer (63) stimulated left primary motor cortex with anodal or sham tDCS twice daily before each training session of intensive naming therapy for 8 days in 2 weeks. A significant enhancement in both trained and untrained words was observed in the anodal group as compared to the sham group. Another study by Darkow et al. (57) explored the impact of anodal stimulation on the left primary motor cortex, however, no significant improvement was observed in accuracy or latency in the naming task. One potential strength of the study was the use of functional magnetic resonance imaging (fMRI) to understand stimulation-induced neural markers. They observed an enhanced brain activation within a larger naming network accompanied by reduced activity in brain regions associated with higher order cognitive control. A 2017 study (59) utilized individualized montages to study stimulaton-induced changes. A more prominent improvement was observed in the number of nouns generated in the stimulation group as compared to the sham. Another study (41) utilizing TMS and tDCS stimulation did not report any significant improvement in tDCS stimulation group as compared to sham stimulation.

**Figure 5:**
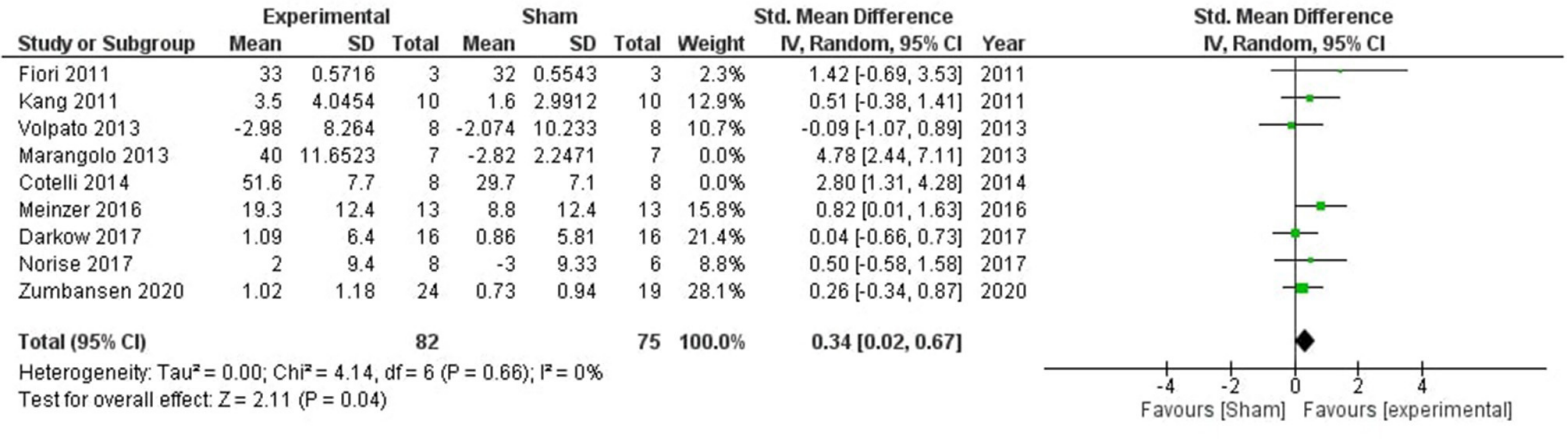
Comparison of the effects of active transcranial stimulation and sham stimulation interventions on aphasia. Two studies were excluded after sensitivity analysis. The results indicated that real stimulation is significantly better than sham stimulation in facilitating the verbal learning (SMD = 0.34; 95% CI = 0.02–0.67; *p* = 0.04).

A significant impact of stimulation was observed in improving aphasic deficits (SMD = 0.84, CI = 0.22–1.47, *p* = 0.008) with high heterogeneity (*I*^2^ = 70%; Supplementary Figure 4). Two studies (50, 52) were removed after sensitivity analysis which reduced the heterogeneity to 0%, and the overall effect of stimulation was still significant (SMD = 0.34, CI = 0.02–0.67, *p* = 0.04; Figure 5).

### Visual Neglect

Sunwoo et al. (49) studied the effects of single-mode and dual-mode anodal stimulation on post-stroke unilateral visuospatial neglect. A significant improvement was observed both in single and dual-mode with the latter showing a more prominent impact. For consistency with other studies, we only used single-mode results in the meta-analysis. Bang and Bong (54) investigated the effect of tDCS and feedback training on visuospatial neglect. A significant improvement was observed in the experimental group as compared to the control group. Another study (53) administered stimulation on visual neglect patients along with prism adaptation training. A significant improvement was observed in the anodal group but no improvement was observed in the cathodal group as compared to the sham. Only anodal stimulation data were pooled in the meta-analysis to be consistent with other studies.

**Figure 6:**
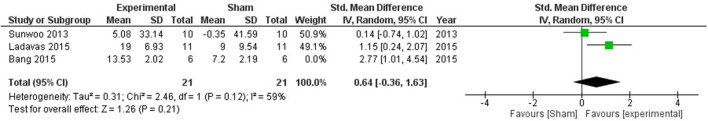
Comparison of the effects of active transcranial stimulation and sham stimulation interventions on visual neglect. One study was excluded after sensitivity analysis. No stimulation related significant effect was observed (SMD = 0.83; 95% CI = −0.49–2.15; *p* = 0.22).

No significant impact of stimulation was observed (SMD = 1.16, CI = −0.09–2.41, *p* = 0.07) with high heterogeneity (*I*^2^ = 73%) (Supplementary Figure 5). One study (54) was removed after sensitivity analysis which reduced the heterogeneity to 59%, and the overall effect of stimulation was still non-significant (SMD = 0.64, CI = −0.036–1.63, *p* = 0.21; Figure 6).

### Risk of Bias Assessment

The risk of bias assessment for the included studies using the Jadad scale is provided in Supplementary Materials.

## Discussion

Transcranical Electrical Stimulation (TES) has emerged as a potential tool to alter brain activity and possibly induce changes in behavior. In case of tDCS the stimulation of any specific brain region results in an enhancement or inhibition of the neuronal activity i.e., anodal stimulation depolarizes the resting state potential, while cathodal stimulation hyperpolarizes it (30). TACS offers another approach to target brain regions in a frequency dependent manner. The idea of stimulating the brain at certain frequencies stems from the fact that different frequencies in the brain represent different neural mechanisms (64) and stimulation of the brain with those frequencies can result in a phenomenon called entrainment in a way that the endogenous brain activity aligns with the applied electric current frequency (19). The current meta-analysis investigated the effects of tES on various cognitive domains including global cognition, attention, working memory, executive functions, aphasia, and visual neglect. Nineteen studies met the criteria for inclusion in this meta-analysis. All the included studies utilized tDCS, and we cannot identify any study using tACS or tRNS.

The meta-analysis showed an acute facilitation effect of tDCS in aphasia and attention, but no improvement was observed in global cognition, working memory, and visual neglect. All the studies on attention, stimulated the DLPFC. The DLPFC is a key node of the dorsal cognitive circuit and is involved in executive functioning and control of cognitive processes (65). Although temporary enhancement of attention was observed, the long-term effects of tES in attention enhancement still need to be investigated with follow-up studies. Most studies on aphasia targeted Broca's area, a key node for speech production (66) or Wernicke's area, which is responsible for speech perception or for word comprehension (67). Stimulation at these regions has shown a reliable effect in promoting the performance of the stroke subjects in verbal learning tasks.

However, there are major limitations in the current meta-analysis. First of all, most of the included studies only evaluated immediate post-stimulation effects without performing any follow-up evaluation. The short-term effect provides us a notion that repetitive stimulation training in longitudinal paradigms may induce plasticity in the brain in the long term, however, this hypothesis can only be verified with further studies evaluating long-term modulatory effects of stimulation. Furthermore, the studies utilized varying number of training sessions. Just like learning any new task requires multiple exposures, tDCS may impact cognition via repeated exposure and overnight consolidation (68). Thus, longitudinal studies must be conducted with follow-up analysis to determine if stimulation can actually improve functional ability in stroke patients with cognitive deficits.

In addition, we observed a high heterogeneity in the results among studies across all cognitive domains. The reason of the heterogeneity could be attributed to several factors, including but not limited to the difference in pathophysiology of the disorder in participants, varying cognitive tests, the number of cognitive training sessions, and most importantly limited number of studies. Furthermore, technical factors linked to tDCS may have played a major role in the variation. Studies have demonstrated that stimulation montage, stimulation duration and intensity, and stimulation type play a critical role in determining the impact of stimulation (33, 69, 70). Some of the studies were removed after sensitivity analysis to reduce the heterogeneity in several cognitive domains, such as one prominent study by Shaker et al. (60) which strongly favored the efficacy of stimulation combined with cognitive training. This particular study focused on immediate offline effects of stimulation during which RehaCom training was provided. The timing and duration of the stimulation are crucial factors determining the impact of stimulation, and it has been reported that offline effects of tDCS might be more pronounced than online effects (71). Furthermore, RehaCom is an intensive rehabilitation training paradigm that has proven effective in improving cognitive functions after stroke (72, 73). The combination of stimulation with an intensive training paradigm like RehaCom might be the best way forward for cognitive rehabilitation. However, further studies need to be conducted to investigate the efficacy of concurrent stimulation with intensive cognitive training paradigms like RehaCom. Furthermore, most studies in the meta-analysis had a very small sample size which can possibly bias the results. Another factor that should be taken into consideration is that the improvement in behavioral performance in laboratory tasks may not directly reflect improvement in functional ability and daily life of the participants (74). Future studies should include assessments to understand if improvement in a specific cognitive domain in a laboratory task is reflected in the daily life of the participants.

All the included studies in the current meta-analysis utilized conventional tDCS, which uses two electrodes, with one acting as a target electrode and the other as a reference electrode, and both of them modulate brain activity in opposite ways. Therefore, it is recommended to put the reference electrode at a location where it can influence brain activity as little as possible (75, 76). However, in some of the included studies, this was not taken into consideration. Furthermore, HD-tES has emerged as a more focal variant that can target cortical brain regions with more accuracy (77). No study in the current meta-analysis utilized HD-tES, which may be utilized in future studies. Furthermore, when it comes to targeting deeper brain structures such as the hippocampus, which is the main hub in memory-related tasks (78) or even anterior cingulate cortex (79) which is responsible for interference control, the existing stimulation devices do not have enough penetration power and even focality to reach these with precision without influences the overlying cortex. Temporal interference stimulation (TIS) is a recently introduced non-invasive methodology to target deeper brain regions with precision without influencing superficial brain structures (80). In TIS, two alternating currents are passed in the brain at a very high frequency in a way that they interact at the target brain region and produce an envelope of difference frequency which modulates the underlying neurons at the envelope frequency. The studies on rodents and simulation models have provided convincing evidence that TIS has the potential to target deeper structures with precision (80, 81). The use of TIS in stroke patients could possibly provide an interesting avenue for cognitive rehabilitation.

## Moving Toward the Standardization of Protocols

The meta-analysis provided some evidence for the efficacy of stimulation methods in improving cognitive deficits in stroke patients. However, all the included studies used apriori determined stimulation location and parameters such as electrode size, stimulation duration, and intensity to stimulate specific brain regions. The lack of mechanistic rationale for determining stimulation location and parameters for each individual makes it impossible to determine if the target brain region was stimulated. Furthermore, using exactly similar parameters may have a different physiological impact on each individual's brain due to differences in individual morphology which further exacerbates reproducibility issues in behavioral results. Thus, the heterogeneous effect of tES in cognitive rehabilitation may be attributed to this mechanistic drawback. Balderston et al. proposed a general workflow of fMRI-guided TMS stimulation (82), describing procedures to apply personalized neuromodulation based on individualized finite element models and fMRI-guided functional localization. We propose that adopting similar protocols on tES would further enhance the efficacy of tES in post-stroke cognitive rehabilitation.

Specifically, the first step involves modeling tES-induced electric field distribution from the individualized volume conductor model (VCM). The VCM is usually generated from high resolution (<1-mm isotropic) structural T1- and T2-weighted MRI. This step does not require expert knowledge thanks to free pipelines available such as SIMNIBS (83) and ROAST (84) in which the electric field simulations on healthy subjects have been well-validated (85). It is important to note that the presence of stroke lesion would affect simulation results' accuracy due to the potential shunting effect at the lesion location (86). Adjusting this effect requires manual labeling of stroke lesions and correction of segmentation results. Lesion masks could be generated by manual delineation from MRI images or automated lesion segmentation pipelines such as LINDA (87). The volumetric segmentation of stroke lesions could be directly incorporated in the VCM for electric field simulation. Electric field simulation would be obtained by solving the Laplace Equation by finite element solver, after the definition of tissue electrical conductivity properties (88). In chronic stroke, stroke lesions could be modeled as cerebrospinal fluid, as suggested in several modeling studies (89). Diffusion weighted images could also be used to model the white matter anisotropy for more accurate simulation results.

The second step is the definition of stimulation target for optimization. Balderston et al. (82) suggest the use of a task-based BOLD activity to localize specific brain functions. Task-based fMRI is widely used to localize the activation foci of cognitive activities. Targeting different cognitive domains requires different tasks. For instance, attention is usually assessed in the classic Flanker Task and Go/No-Go tasks, which mainly involve the activation of the frontal lobe (90, 91). Other tasks like color-word stroop tasks, and n-back working memory tasks could be utilized to localize regions related to executive functions and working memory (92, 93). The fMRI activation map could be projected to the volume conductor model for the subsequent optimization procedure. Identifying individual activation foci is essential because of significant inter-individual state-dependent brain activity (94, 95).

After the definition of stimulation target, the last step is to optimize electrode placement. In the ideal scenario, only the target would be stimulated, while other regions remain unaffected. Therefore, the goal of optimization is maximizing the electric field on the target while minimizing off-target stimulations. With the provided electrode' size and geometry, optimization could be performed by identifying the electrode's location based on the international 10/20 system and their corresponding intensities by genetic algorithm. It is important to note that tDCS is orientation-dependent (96). The direction in which the electric field is optimized requires more investigations. For instance, normal electric field component was found to influence corticospinal excitability (97), whereas electric field magnitude could also be used to predict actual neurophysiological measures (98). Although the best way to define the objective functions for optimization to provide maximal stimulation to the defined target and generate a significant neuromodulation effect needs further exploration.

Existing literature on brain stimulation is primarily based on the hit and trial approach, which does not guarantee stimulation of the target brain region. Here we briefly outlined the procedure that could be adopted in future studies to make stimulation studies more consistent, comparable, and reproducible and ultimately make tES acceptable in the scientific community as a reliable therapeutic tool.

## Conclusion

The meta-analysis favors the efficacy of tDCS in an acute improvement in aphasia and attention. However, the results should be interpreted with caution due to methodological and technical limitations in the included studies. We conclude that stimulation paradigms have a beneficial effect in improving some aspects of cognition, but they cannot be generalized across different cognitive domains. Furthermore, the evidence of their efficacy is weak and standardized stimulation protocols with improved experimental design and follow-up analysis may provide us a better understanding of the clinical effectiveness of stimulation methods in cognitive rehabilitation.

## Data Availability Statement

The original contributions presented in the study are included in the article/Supplementary Material, further inquiries can be directed to the corresponding author/s.

## Author Contributions

AK and KY devised a search strategy, searched articles on different databases, and screened the studies based on titles and abstracts. AT and NA extracted relevant information from all the included studies. CT and S-CB assessed methodological quality and the risk of bias in each of the included studies. RT supervised the whole study and was consulted in case of a disagreement about any study. All authors contributed to the article and approved the submitted version.

## Conflict of Interest

The authors declare that the research was conducted in the absence of any commercial or financial relationships that could be construed as a potential conflict of interest.

## Publisher's Note

All claims expressed in this article are solely those of the authors and do not necessarily represent those of their affiliated organizations, or those of the publisher, the editors and the reviewers. Any product that may be evaluated in this article, or claim that may be made by its manufacturer, is not guaranteed or endorsed by the publisher.
