# Supplementary material for: Can Transcranial Electrical Stimulation Facilitate Post-stroke Cognitive Rehabilitation? A Systematic Review and Meta-Analysis
Source: Front Rehabil Sci. 2022 Feb 10;3:795737. doi: 10.3389/fresc.2022.795737 (PMC9397778; doi:10.3389/fresc.2022.795737)
Supplement: Supplementary file 1 [file Data_Sheet_1.docx]

**Supplementary Materials: Can transcranial electrical stimulation facilitate post-stroke cognitive rehabilitation. A systematic review and meta-analysis**

**Detailed search strategy:**

Stroke

1. cerebrovascular disease/ or exp basal ganglion hemorrhage/ or exp brain hematoma/ or exp brain hemorrhage/ or exp brain infarction/ or exp brain ischemia/ or exp carotid artery disease/ or cerebral artery disease/ or exp cerebrovascular accident/ or exp intracranial aneurysm/ or exp occlusive cerebrovascular disease/ or stroke patient/

2. (stroke or poststroke or post-stroke or cerebrovasc$ or brain vasc$ or cerebral vasc$ or cva$ or apoplex$ or SAH).tw.

3. ((brain$ or cerebr$ or cerebell$ or intracran$ or intracerebral) adj5 (isch?emi$ or infarct$ or thrombo$ or emboli$ or occlus$)).tw.

4. ((brain$ or cerebr$ or cerebell$ or intracerebral or intracranial or subarachnoid) adj5 (haemorrhage$ or hemorrhage$ or haematoma$ or hematoma$ or bleed$)).tw.

5. hemiparesis/ or hemiplegia/ or paresis/

6. (hemipleg$ or hemipar$ or paresis or paretic or hemineglect or hemi-neglect or ((unilateral or spatial or hemi?spatial or visual) adj5 neglect)).tw.

7. or/1-6

Cognitive disorder

8. cognition disorder$/ or neurobehavioral manifestation$/ or confusion/ or memory disorder$/ or mental process$/ or cognition/ or comprehension/ or learning/ or generalization psychology/ or transfer psychology/ or perception/ or thinking/ or concept formation/ or judgment/ or problem solving/ or perceptual disorder$/ or arousal/ or orientation/ or attention/ or awareness/ or memory/ or recognition psychology/ or algorithm$/ or impulsive behavior/ or neuropsychological test$/ or metacognition/

9. (agnosia or amnesia or confusion or inattention).tw.

10. (cognit$ or arous$ or orientat$ or attention$ or concentrate$ or memor$ or recall or percept$ or think$ or sequenc$ or algorithm$ or judgement$ or judgment$ or awareness or problem solving or generalization or transfer or comprehension or learning).tw.

11.(disorder$ or declin$ or dysfunct$ or impair$ or deficit$ or ability$ or problem$).tw.

12. 10 and 11

13.(dysexecutive syndrome$ or dysexecutive function$ or mental process$ OR impulsive behavior$ or impulsive behaviour$ or executive function or executive dysfunction or front striatal dysfunction).tw.

14. 8 or 9 or 12 or 13

Transcranial electrical stimulation

15. transcranial electrical stimulation/ or transcranial direct current stimulation/ or transcranial direct-current stimulation/ or tDCS / or tES/ or transcranial alternating current stimulation/ or transcranial alternating-current stimulation/ or tACS/ or transcranial random noise stimulation/ or tRNS/

16. electrostimulation therapy/ or nerve stimulation/ or electrostimulation/

17. electrode/

18. (transcranial adj5 direct current adj5 stimulation).tw.

19. (transcranial adj5 DC adj5 stimulation).tw.

20. (transcranial adj5 electric$ adj5 stimulation).tw.

21. (tDCS or A-tDCS or C-tDCS or S-tDCS or electrode$ or anode or anodes or anodal or cathode or cathodes or cathodal).tw.

22. (transcranial adj5 alternating current adj5 stimulation).tw.

23. (transcranial adj5 AC adj5 stimulation).tw.

24. or/15-23

RCT

25. Randomized Controlled Trial/ or "randomized controlled trial (topic)"/

26. Randomization/ or Randomisation/

27. Controlled clinical trial/ or "controlled clinical trial (topic)"/

28.clinical trial/ or "clinical trial (topic)"/ or phase 1 clinical trial/ or phase 2 clinical trial/ or phase 3 clinical trial/ or phase 4 clinical trial/

29. Crossover Procedure/

30. Double Blind Procedure/

31. Single Blind Procedure/ or triple blind procedure/

32. placebo/ or placebo effect/

33. (random$ or RCT or RCTs).tw.

34. (placebo$ or sham).tw.

35. control group/ or controlled study/

36. or/ 25-35

7 and 14 and 24 and 36

**Supplementary figures:**


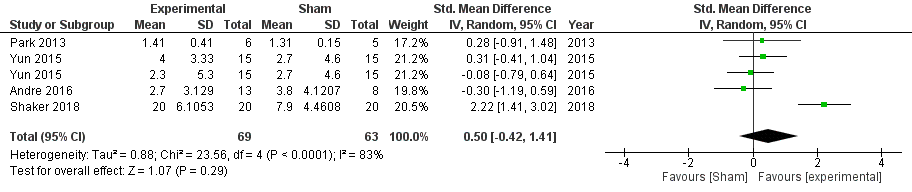


Figure S1: tES effects on global cognition before sensitivity analysis


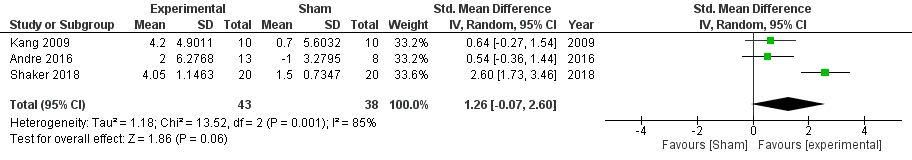


Figure S2: tES effects on attention before sensitivity analysis

***
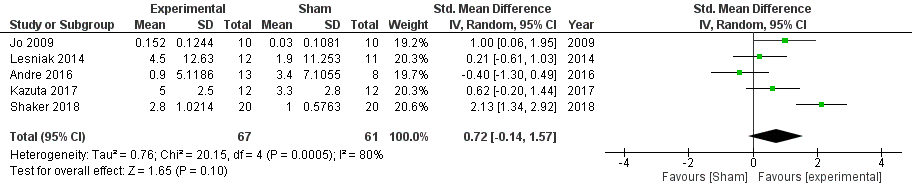
***

Figure S3: tES effects on working memory before sensitivity analysis


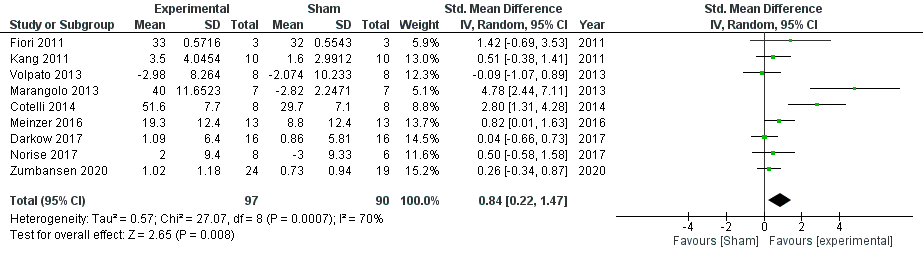


Figure S4: tES effects on aphasia before sensitivity analysis


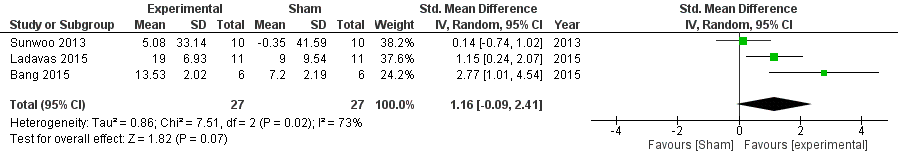


Figure S5: tES effects on visual neglect before sensitivity analysis

**Supplementary Tables:**

Table S1: Modified Jadad scores of the included studies.

| **Author** | **Was the**  **research**  **described as**  **randomized?**  **#** | **Was the**  **approach of**  **randomization appropriate?** | **Was the**  **research**  **described**  **as blinding?**  **#** | **Was the**  **approach of**  **blinding**  **appropriate?*** | **Was there a**  **presentation of withdrawals**  **and**  **dropouts?#** | **Was there a**  **presentation of the**  **inclusion/exclusion criteria?#** | **Was the**  **approach**  **used to**  **assess**  **adverse**  **effects**  **described?**  **#** | **Was the**  **approach**  **of**  **statistical**  **analysis**  **described?**  **#** | **Total** |
| --- | --- | --- | --- | --- | --- | --- | --- | --- | --- |
| Kang et al. 2009[34] | 1 | 0 | 1 | 1 | 0 | 1 | 0 | 1 | 5 |
| Jo et al. 2009[37] | 1 | 0 | 0.5 | 1 | 0 | 1 | 1 | 1 | 5.5 |
| Fiori et al. 2010[77] | 1 | 0 | 1 | 1 | 0 | 1 | 0 | 1 | 5 |
| Kang et al. 2011[40] | 0 | 0 | 1 | 1 | 0 | 1 | 0 | 1 | 4 |
| Volpato et al. 2013[41] | 0 | 0 | 0 | 0 | 0 | 1 | 1 | 1 | 3 |
| Sunwoo et al. 2013[78] | 1 | 0 | 1 | 1 | 0 | 1 | 0 | 1 | 5 |
| Marangolo et al. 2013[42] | 0 | 0 | 0 | 0 | 0 | 1 | 0 | 1 | 4 |
| Park et al. 2013[79] | 0 | 0 | 1 | 1 | 0 | 1 | 0 | 1 | 4 |
| Cotelli et al. 2014[43] | 1 | 0 | 0.5 | 0 | 0 | 1 | 0 | 1 | 3.5 |
| Ladavas et al. 2015[50] | 1 | 1 | 1 | 1 | 0 | 1 | 1 | 1 | 7 |
| Yun et al. 2015[31] | 1 | 0 | 1 | 1 | 0 | 1 | 0 | 1 | 5 |
| Bang et al. 2015[80] | 1 | 1 | 0 | 0 | 0 | 0 | 0 | 1 | 3 |
| Andre et al. 2016[81] | 1 | 0 | 0.5 | 0 | 1 | 1 | 1 | 1 | 5.5 |
| Meinzer et al. 2016[82] | 1 | 1 | 1 | 1 | 1 | 1 | 1 | 1 | 8 |
| Norise et al. 2017[46] | 0 | 0 | 0 | 0 | 0 | 1 | 0 | 1 | 2 |
| Darkow et al. 2017[45] | 1 | 1 | 1 | 1 | 1 | 1 | 1 | 1 | 8 |
| Kazuta et al. 2017[38] | 1 | 0 | 0.5 | 0 | 0 | 1 | 0 | 1 | 3.5 |
| Shaker et al. 2018[33] | 1 | 0 | 0.5 | 0 | 0 | 1 | 0 | 1 | 3.5 |
| Zumbansen et al. 2020[47] | 1 | 1 | 0.5 | 0 | 1 | 1 | 1 | 1 | 6.5 |
